# How Performing Chest Compressions Influences Mental Arithmetic Capabilities: A Randomized Cross-Over Trial

**DOI:** 10.3390/jcm14103366

**Published:** 2025-05-12

**Authors:** Caroline Holaubek, Mathias Maleczek, Maximilian Scheidl, Anna Maleczek, Nikolaus Frimmel, Julius Goschin, Bernhard Roessler

**Affiliations:** 1 Clinical Division of General Anaesthesia and Intensive Care Medicine, Department of Anaesthesia, Intensive Care Medicine and Pain Medicine, Medical University of Vienna, 1090 Vienna, Austria; 2 Academic Simulation Center Vienna, 1090 Vienna, Austria; 3 Department Health Sciences, University of Applied Sciences FH Campus Vienna, 1100 Vienna, Austria

**Keywords:** multi-tasking, resuscitation, chest compression, simulation, training

## Abstract

**Background/Objectives**: Performing cardiopulmonary resuscitation (CPR) is cognitively demanding, often requiring helpers to perform cognitive and manual tasks simultaneously. While the human brain primarily switches between tasks rather than processing them simultaneously, it remains unclear whether performing repetitive, monotonous manual tasks, such as chest compressions, affects cognitive performance. This study aimed to assess the impact of chest compressions on mental arithmetic performance. **Methods**: In a randomized crossover trial, healthy participants trained in advanced life support (physicians, nurses, and paramedics) completed the Paced Auditory Serial Addition Test (PASAT) under two conditions: with or without performing chest compressions on a manikin. The primary outcome was the number of correct PASAT answers. Secondary outcomes included workload assessment using the NASA Task Load Index (TLX) and chest compression (CC) performance. The trial was registered at clinicaltrials.gov and approved by the local ethics committee. **Results**: Thirty-eight participants were included. The number of correct PASAT responses was significantly lower during chest compressions compared to the control (36.5 vs. 41; *p* < 0.01). NASA TLX values were significantly higher in the chest compression condition, indicating increased perceived workload. Chest compression performance showed statistically significant differences between a phase of just chest compressions and during the PASAT, especially increased levels of incomplete recoil. **Conclusions**: This study demonstrates that even a simple repetitive motor task like chest compressions impairs cognitive task performance significantly. Furthermore, multitasking was shown to decrease chest compression quality. These findings strongly highlight the importance of effective task allocation and minimizing multitasking during CPR to optimize performance and thereby patient outcomes.

## 1. Introduction

Human cognition has fundamental limitations when it comes to multitasking. Despite the common belief that we can efficiently handle multiple tasks at once, research has consistently shown that the brain does not truly multitask—rather that it rapidly switches between tasks, often with significant cognitive costs [1,2,3]. This limitation has critical implications in high-stakes environments such as resuscitation, where rapid decision-making and precise execution are essential [4,5]. Just as using a phone while driving leads to decreased reaction times and increased error rates [6], attempting to manage multiple cognitive and motor tasks simultaneously during resuscitation may compromise patient outcomes [5,7]. Studies on attention and cognitive load have demonstrated that task-switching not only increases mental effort but also introduces delays and errors, particularly in complex and time-sensitive situations in medicine [7,8,9].

In resuscitation scenarios, healthcare providers must perform a variety of tasks, often taking on several tasks at the same time, including performing chest compressions, communicating with the team, and executing critical interventions like vascular access or advanced airway management—all within a short timeframe [10,11]. Urgency makes multitasking appealing and, at times, seemingly necessary. However, research into human factors and medical performance suggests that structured task management, delegation, and cognitive offloading are more effective strategies to maintain overall performance and minimize errors [12,13,14].

A particularly relevant question is whether mental tasks can be performed concurrently with chest compressions. One relevant mental task is mental arithmetic, as this is relevant for multiple parts of resuscitation, like dose calculation, especially during paediatric resuscitation. Furthermore, mental arithmetic seems to be a good surrogate for other mental tasks. In low-resource settings, such as prehospital resuscitations, it may be tempting to assign cognitive tasks to the team member performing chest compressions, as this seems to be a repetitive, simple task [15]. In a simulated virtual reality setting with first-year residents performing basic life support, including bag-mask ventilation, a recent study has demonstrated that the quality of chest compressions declines when additional cognitive tasks, such as mental arithmetic, are performed simultaneously [16]. The participants presumably had little to no experience in providing real-life advanced cardiac life support, leaving the question of whether more experienced providers are less cognitively burdened by chest compressions. Furthermore, this trial used virtual reality headsets, which could lead to increased mental load itself [17]. To address those factors, a trial was conducted to examine the impact of chest compressions on mental arithmetic performance among highly trained clinicians responsible for resuscitations in their clinical practice. To further reduce possible other factors, chest compression without any other distractions was studied.

## 2. Materials and Methods

### 2.1. Study Design

A randomized cross-over trial was conducted at the Academic Simulation Centre Vienna according to the Helsinki Declaration after approval by the Medical University of Vienna’s Ethics Committee (EK 1453/2024, Approval date: 29 May 2024). The trial was registered at clinicaltrials.gov (NCT06869642, date: 10 March 2025).

### 2.2. Inclusion/Exclusion Criteria

Medical professionals trained in CPR (anaesthesiologists, emergency physicians, internal medicine physicians, nurses, and paramedics) above the age of 18 were included. Informed consent was obtained and signed by all subjects involved in the study. They had to have attended a CPR training in the previous four years and had to feel well and rested at the time of inclusion. Pregnant volunteers were excluded.

### 2.3. Variables

To assess multitasking abilities, the Paced Auditory Serial Addition Test (PASAT) was utilized. The PASAT is a widely used neuropsychological test designed to evaluate cognitive processing speed, working memory, and sustained attention [18]. During the test, the participants hear a series of single-digit numbers presented at fixed two-second intervals and must continuously add each new number to the one immediately preceding it, reporting the sum aloud. The PASAT is known to impose a high cognitive load, making it an effective tool for assessing mental workload and stress responses in time-sensitive tasks. Therefore, it is ideal for testing multitasking [19].

The Task Load Index (TLX) was collected after each round. This index was shown to reflect the mental load of tasks in a reliable way [20,21,22,23]. Furthermore, the following details of chest compressions were collected: chest compression rate, chest compression depth, and the amount of leaning.

### 2.4. Methodology

The trial was conducted in a controlled simulation environment. All test instructions were standardized and delivered via the room’s audiovisual system (SimStation, Vienna, Austria). The entire trial was recorded using the integrated audiovisual unit for further analysis. To simulate a realistic resuscitation scenario, adult CPR manikins were used, primarily the Gaumard HAL 3201 (Gaumard Scientific, Miami, FL, USA), which was switched to a Laerdal Resusci Anne QCPR (Laerdal, Norway) after technical difficulties with recording the chest compression parameters. Performance data were collected using the Laerdal SimPad system (Laerdal, Norway) or the Gaumard Uni 2.* software (Gaumard Scientific, FL, USA), ensuring accurate tracking of CPR metrics like chest compression rates, depth, and incomplete recoil. The analysis was conducted using Python 3.8 [24]. To comply with the current standards of CPR, a metronome (100 bpm) was mandatory for all participants, who were asked to perform continuous high-quality chest compressions only. The PASAT started after 30 s of chest compressions, resulting in a total of 2.5 min of chest compressions. Each participant completed two different rounds of PASAT. After each round, the participants were asked to fill out the NASATLX. A picture of the setup used during the trial is shown in the Supplemental Figure S1.

### 2.5. Randomization

**Figure 1:**
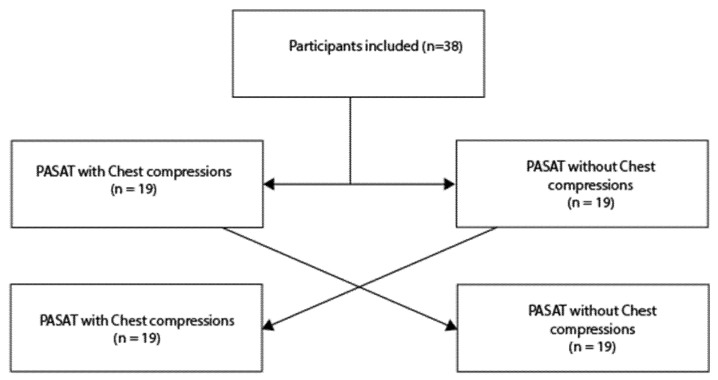
Figure shows the study’s crossover design.

The participants were allocated either to the PASAT with or without conducting chest compressions and crossed over afterwards to compensate for any learning of the test (Figure 1).

The second phase of data collection was conducted directly following the first as soon as the participant felt ready to do so. Randomization was conducted using a block randomization calculated in Python 3.8. with blocks of ten persons and a 50/50 manner. Opaque envelopes with participant IDs on the outside contained the information about randomization.

### 2.6. Outcome

The number of correct answers on the PASAT was used as the primary outcome measure. The null hypothesis stated that there would be no difference in the number of correct answers between the two groups.

### 2.7. Sample Size Calculation

To detect a mean difference of 1 (SD: 2), 34 participants were needed for an alpha of 0.05 and a beta of 0.8. (paired *t*-test) With an expected drop-out rate of 10%, 38 participants were planned to be included.

### 2.8. Statistical Analysis

Demographic details were presented using descriptive statistics. Age was collected in age groups of a 5-year span to comply with local regulations of the data safety board. A paired *t*-test was used to test the primary hypothesis. Regarding the secondary outcomes, a paired *t*-test was used to show differences in NASA-TLX. Regarding the characteristics of chest compressions (depth, rate, and incomplete recoil), mixed linear models were used to account for the different numbers of observations. A 30 s baseline was compared to two minutes of chest compressions during the PASAT.

## 3. Results

A total of 38 participants were included in this trial. The cohort comprised 17 (45.0%) females and 21 (55.0%) males, most of them between 30 and 40 years old. Regarding physicians, both anesthesiologists and internal medicine doctors were included. All of those were prehospital emergency physicians (certified or in training). Additional demographic details can be found in Table 1. All 38 completed the trial and could be included in the main analysis. A CONSORT flowchart can be found in the Supplementary Materials.

**Figure 2:**
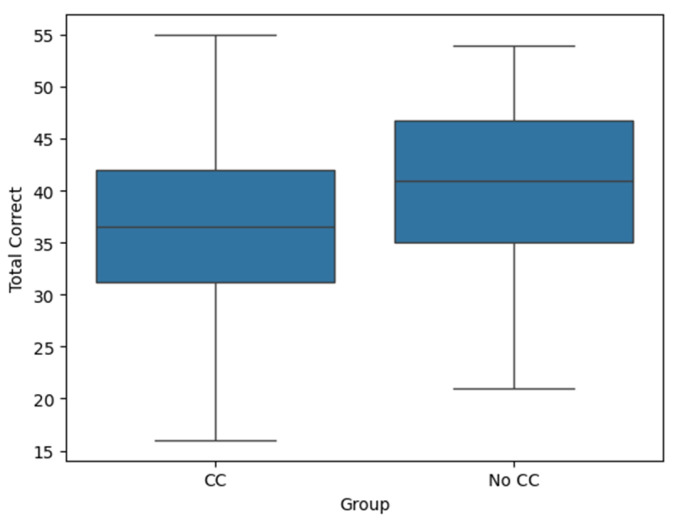
Figure shows boxplots comparing the number of correct answers in the PASAT between the chest compression (CC) and no chest compression group (No CC).

The median number of correct answers on the PASAT was significantly lower during performance of chest compressions (36.5, range: 16–55) compared to not performing chest compressions (41, range: 21–54) (*p* < 0.01). On average, the participants scored 3.05 fewer correct answers during chest compressions compared to the PASAT alone (95% CI: −5.18 to −0.93). The effect size, calculated as Cohen’s *d*, was −0.472, indicating a moderate negative effect of chest compressions on performance (Figure 2).

**Figure 3:**
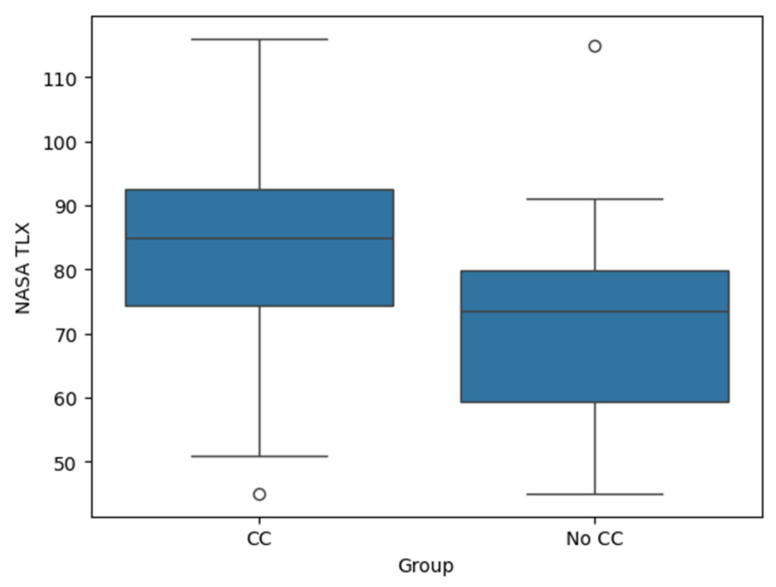
Figure shows boxplots comparing the NASA Task Load Index (NASA TLX) between the chest compression (CC) and no chest compression groups (No CC).

The NASA-TLX scores were significantly higher during the PASAT while performing chest compressions (median TLX score: 85, range: 45–116) compared to the PASAT alone (median: 73.5, range: 45–115), *p* < 0.001 (Figure 3). On average, the participants reported a NASA TLX 12.3 points higher during chest compressions compared to the PASAT alone (95% CI: 7.0 to 17.6). The effect size, calculated as Cohen’s *d*, was 0.768, indicating a medium-to-large effect of chest compressions on the NASA TLX.

**Figure 4:**
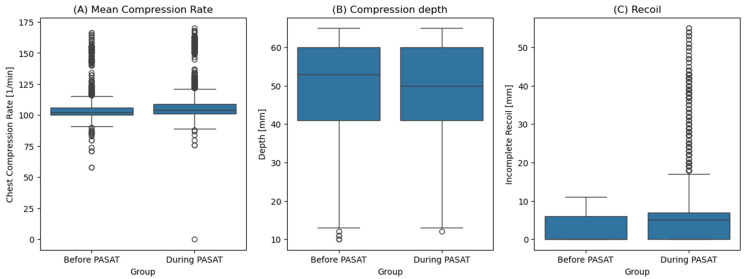
Figure shows boxplots comparing the participants’ chest compression performance. The baseline was recorded at the beginning of compressions, while the test phase was recorded during the PASAT. (**A**) Mean rate of chest compressions, (**B**) compression depth, (**C**) incomplete recoil during chest compressions.

Due to technical difficulties collecting data on chest compression quality, five participants had to be excluded from the analysis of chest compression characteristics (three in the CC first group and two in the PASAT without CC first group). For the remaining 33 participants, the chest compression characteristics differed significantly between performing the PASAT and baseline (*p* < 0.01 for all parameters). The median compression rate increased from 102/min (baseline) to 104/min (PASAT). The median compression depth decreased from 53 mm to 50 mm, and the median incomplete recoil depth increased from 0 mm to 5 mm (Figure 4, Table 2).

## 4. Discussion

This trial included 38 well-trained healthcare professionals and assessed the multitasking abilities during chest compressions using the Paced Auditory Serial Addition Test (PASAT). The number of correct mental calculations was significantly lower during chest compressions compared to concentrating on the calculations alone.

The reduction in the number of correct answers on the PASAT during chest compressions highlights the cognitive burden associated with performing manual tasks in high-stakes, time-sensitive environments like resuscitation. These findings align with the broader literature on cognitive load and task switching, which suggests that, while humans often attempt to multitask, our cognitive system is not designed to perform multiple complex tasks simultaneously without performance degradation [4,7,8,25].

These results therefore have implications for both clinical practice and training, where health care providers can be trained to perform chest compressions only and speak up for a better distribution of the cognitive load, when assigned additional tasks [7,16]. Especially in simulation and training, facilitators should emphasize the importance of minimizing cognitive load and recognizing the limited capacity to perform additional tasks while delivering chest compressions. During resuscitation, it is advisable for team leaders to distribute the workload in a way that prevents the need for multitasking [26].

Furthermore, the NASA-TLX scores in our study provided additional insights into the perceived mental load. Participants reported significantly higher cognitive workload when performing chest compressions alongside the PASAT, indicating that multitasking in resuscitation settings not only impairs performance but also increases the perceived difficulty and stress of the task. This leads to impaired team performance and possibly even to increased mortality [27,28]. This finding is in line with the literature on cognitive load, which has shown that tasks that require sustained attention and high cognitive resources (such as the PASAT) are particularly sensitive to interference from concurrent tasks [5,7,16].

Training has an important role in CPR, with short intervals between the training sessions seeming beneficial [29,30,31]. The participants were recruited in a simulation center, suggesting a positive attitude toward training. This is reflected in the median time since their last advanced cardiac life support training, which was only four months. Therefore, it could be assumed that the included participants should not be distracted by performing chest compressions. Their generally high chest compression performance further supports this assumption. The interquartile range (25th–75th percentile) of the chest compression rate remained within the 100–120/min range recommended by the European Resuscitation Council (ERC) and the American Heart Association (AHA) guidelines [32,33]—possibly due to the metronome used as suggested by those guidelines. Values outside of this range in the before PASAT group can possibly be attributed to the fact that the participants were still adjusting to the manikin during the first compression of the study. The median chest compression depth was within the recommended 5–6 cm both during the baseline and during the PASAT.

While the guidelines emphasize the importance of avoiding incomplete recoil during compressions, increased incomplete recoil was observed in this cohort and further intensified during the PASAT [32,33]. While differences in compression rate and depth were statistically significant, they were likely clinically irrelevant. However, the increased incomplete recoil depth may pose a significant risk, as it can impair ventricular filling and subsequently reduce cardiac output [34]. These findings highlight the potential impact of cognitive distraction on the quality of chest compressions during CPR. Notably, the capacity for self-correction appears to be limited under conditions of cognitive overload. As such, engaging in cognitive tasks while performing chest compressions may pose a significant risk to patient outcomes during cardiac arrest, emphasizing the need to minimize multitasking during the performance of chest compressions.

### Limitations

This study has several limitations that should be considered when interpreting the results. First, the participants were mainly recruited in a simulation center. Thus, they were likely highly motivated and well-trained in resuscitation techniques. This may limit the generalizability of the findings to broader populations, including less experienced providers or those with varying levels of training. Second, the study was conducted in a controlled simulation environment and not in an emergency situation with a human patient, where additional stressors, external distractions, team relationships, and physical fatigue could further influence task performance. Third, although the PASAT was used as a surrogate for complex cognitive tasks, it may not fully represent the diverse mental demands encountered during actual CPR, such as decision-making under pressure and team communication. While the PASAT is a validated tool for assessing processing speed and working memory, it does not capture other critical cognitive components relevant to real-life resuscitation, including situational awareness and interpersonal coordination [20,21,22,23]. Future studies should incorporate broader assessments to better reflect the complex cognitive environment of clinical practice.

Another limitation is the exclusion of five participants from the analysis of chest compression characteristics. While the trial was started using a Gaumard HAL 3201 manikin, it had to be replaced with a Laerdal Resusci Anne after the second participant due to the unreliable chest compression recordings. (Both participants had to be excluded from the secondary analysis.) This problem occurred a lot less frequently with the Laerdal mannequin, so only three more probands had to be excluded.

Another potential limitation is the immediate sequential assignment of participants to both conditions. As only one condition involved physical activity, a washout phase was not implemented, which may have introduced a learning effect in the PASAT. However, this was deemed acceptable, given the crossover study design.

Although all participants had routine involvement in resuscitations as part of their clinical duties, the individual level of experience with real-life resuscitations was not systematically recorded. This may have influenced both cognitive and motor performance during the tasks, as more experienced providers may be better equipped to manage multitasking and stress. No feedback about the chest compression depth was available to the participants during the test, although modern defibrillators can provide this feedback. This was conducted in order to reduce the concomitant distractions and prevent an additional increase in cognitive load.

One possible risk of simulation-based research is potentially distressing simulations. Given the importance of participant well-being, it was ensured that a senior simulation facilitator was always available to provide immediate debriefing and support in the unlikely event of distress, and no such incidents occurred during the trial.

This trial demonstrated that mental arithmetic performance declines during the performance of chest compressions. Further research is needed to identify potential interventions that may prevent healthcare professionals from attempting to multitask in such critical situations. Additionally, it would be valuable to investigate the amount and type of training required or the technological assistance necessary to mitigate the cognitive impact of performing chest compressions on tasks such as mental arithmetic.

## 5. Conclusions

This study demonstrates that even a relatively simple motor task, such as performing chest compressions, significantly impairs cognitive task performance, as measured by the Paced Auditory Serial Addition Test (PASAT). Furthermore, chest compression quality also declined during concurrent cognitive task performance. These findings highlight the importance of minimizing multitasking and ensuring effective task allocation during cardiopulmonary resuscitation to optimize both provider performance and patient outcomes.

## Figures and Tables

**Table 1:** Demographic details. (IQR: 0.25 percentile, 0.75 percentile), CC: chest compressions. The CC first group was allocated to take the PASAT while performing chest compressions first, while the No CC first group took the PASAT without chest compressions first.

	Overall	CC First Group	No CC First Group
Age group (%)			
20–25	5 (13.2%)	3 (15.8%)	2 (10.5%)
25–30	8 (21.1%)	5 (26.3%)	3 (15.8%)
30–35	11 (28.9%)	5 (26.3%)	6 (31.6%)
35–40	11 (28.9%)	5 (26.3%)	6 (31.6%)
40–45	1 (2.6%)	0	1 (5.3%)
50–55	1 (2.6%)	0	1 (5.3%)
55–60	1 (2.6%)	1 (5.3%)	0
Female Sex (%)	17 (44.7%)	7 (36.8%)	10 (52.6%)
Male Sex (%)	21 (55.3%)	12 (63.2%)	9 (47.4%)
Specialty (%)			
Physician (Anaesthesia)	17 (44.7%)	6 (31.6%)	11 (57.9%)
Physician (Internal Medicine)	7 (18.4%)	4 (21.1%)	3 (15.8%)
Nurse	7 (18.4%)	4 (21.1%)	3 (15.8%)
Paramedic	7 (18.4%)	5 (26.3%)	2 (10.5%)
Time since last CPR Training [month] (IQR)	4.3 (3.0, 7.1)	4.3 (1.9, 5.6)	4.4 (3.3, 12.5)

**Table 2:** The table presents the median and interquartile range (25th to 75th percentile) for compression depth, mean compression rate, and recoil.

	Before PASAT	During PASAT
Compression Depth [mm]	53.0, (41.0, 60.0)	50.0, (41.0, 60.0)
Mean Compression Rate [/min]	102.0, (100.0, 106.0)	104.0, (101.0, 109.0)
Recoil [mm]	0.0, (0.0, 6.0)	5.0, (0.0, 7.0)

## Data Availability

The data presented in this study are available upon request from the corresponding author due to privacy reasons.

## Supplementary Materials

The following supporting information can be downloaded at: https://www.mdpi.com/article/10.3390/jcm14103366/s1, Figure S1: Schematic representation of the experimental setup used in this trial. (a) CPR manikin positioned on the floor, (b) participant performing resuscitation, (c) simulation room, (d) One-way mirror, (e) control room, (f) study personnel operating the audiovideo-system and the PASAT readings.

## Abbreviations

The following abbreviations are used in this manuscript:

PASAT	Paced Auditory Serial Addition Test
CC	Chest compression
NASA-TLX	National Aeronautics and Space Administration Task Load Index
CPR	Cardiopulmonary resuscitation
ERC	European Resuscitation Council
AHA	American Heart Association
