# Supplementary material for: How Performing Chest Compressions Influences Mental Arithmetic Capabilities: A Randomized Cross-Over Trial
Source: J Clin Med. 2025 May 12;14(10):3366. doi: 10.3390/jcm14103366 (PMC12112591; doi:10.3390/jcm14103366)
Supplement: Supplementary file 1 [file jcm-14-03366-s001.zip › jcm-3610501-supplementary.pdf]

Supplemental Figure 1

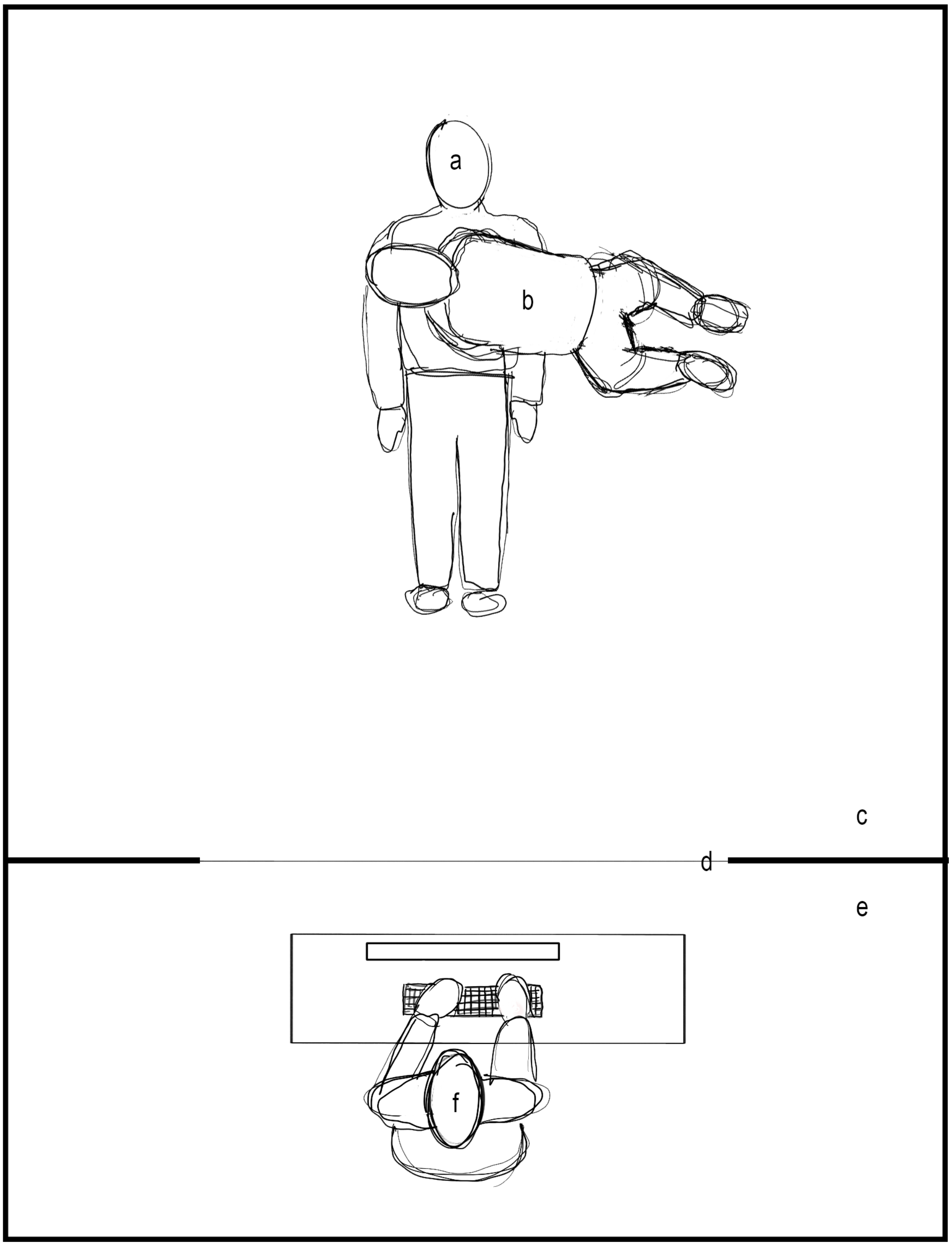

Supplemental Figure 1: Schematic representation of the experimental setup used in this trial. *(a)* CPR manikin positioned on the floor, *(b)* participant performing resuscitation, *(c)* simulation room, *(d)* One-way mirror, *(e)* control room, *(f)* study personnel operating the audiovideo-system and the PASAT readings.
